# Self-Reported Truck Traffic on the Street of Residence and Symptoms of Asthma and Allergic Disease: A Global Relationship in ISAAC Phase 3

**DOI:** 10.1289/ehp.0800467

**Published:** 2009-07-20

**Authors:** Bert Brunekreef, Alistair W. Stewart, H. Ross Anderson, Christopher K.W. Lai, David P. Strachan, Neil Pearce, the ISAAC Phase 3 Study Group

**Affiliations:** 1 Institute for Risk Assessment Sciences and Julius Center for Health Sciences and Primary Care, University Medical Center Utrecht, Utrecht, the Netherlands; 2 School of Population Health, Faculty of Medical and Health Sciences, University of Auckland, Auckland, New Zealand; 3 Division of Community Health Sciences, St. George’s, University of London, London, United Kingdom; 4 Department of Medicine and Therapeutics, Chinese University of Hong Kong, Hong Kong, People’s Republic of China; 5 Center for Public Health Research, Massey University, Wellington, New Zealand

**Keywords:** air pollution, asthma, eczema, rhinitis, truck traffic

## Abstract

**Background:**

Associations between traffic pollution on the street of residence and a range of respiratory and allergic outcomes in children have been reported in developed countries, but little is known about such associations in developing countries.

**Methods:**

The third phase of the International Study of Asthma and Allergies in Childhood (ISAAC) was carried out in 13- to 14-year-old and 6- to 7-year-old children across the world. A question about frequency of truck traffic on the street of residence was included in an additional questionnaire. We investigated the association between self-reported truck traffic on the street of residence and symptoms of asthma, rhinoconjunctivitis, and eczema with logistic regression. Adjustments were made for sex, region of the world, language, gross national income, and 10 other subject-specific covariates.

**Results:**

Frequency of truck traffic on the street of residence was positively associated with the prevalence of symptoms of asthma, rhinoconjunctivitis, and eczema with an exposure–response relationship. Odds ratios (95% confidence intervals) for “current wheeze” and “almost the whole day” versus “never” truck traffic were 1.35 (1.23–1.49) for 13- to 14-year-olds and 1.35 (1.22–1.48) for 6- to 7-year-olds.

**Conclusions:**

Higher exposure to self-reported truck traffic on the street of residence is associated with increased reports of symptoms of asthma, rhinitis, and eczema in many locations in the world. These findings require further investigation in view of increasing exposure of the world’s children to traffic.

Living near busy roads has been associated with increased respiratory symptoms and sensitization rates and with reduced lung function in children (Annesi-Maesano et al. 2007; Brauer et al. 2002, 2006, 2007; Brunekreef et al. 1997; Chang et al. 2009; Ciccone et al. 1998; Dales et al. 2008; Escamilla-Nunez et al. 2008; Gauderman et al. 2004, 2005, 2007; Gehring et al. 2002; Hirsch et al. 1999; Janssen et al. 2003; Jerrett et al. 2008; Karr et al. 2009; Kim et al. 2004, 2008; Mortimer et al. 2008; Oftedal et al. 2009; van Vliet et al. 1997; Venn et al. 2000, 2001, 2005; Wilhelm et al. 2008). One study (Oftedal et al. 2009) failed to find a positive association. Early studies relied on questionnaires to ascertain exposure as well as symptoms, making it difficult to exclude responder bias as the explanation for the associations that were found (Duhme et al. 1996; Weiland et al. 1994). The validity of the questionnaire-based exposure assessment has been addressed (Ciccone et al. 1998), and later studies have relied more on objective ascertainment of exposure as well as disease (Brauer et al. 2002; Brunekreef et al. 1997; Gehring et al. 2002; Janssen et al. 2003). These latter studies have shown that several air pollution components are clearly elevated in air near busy roads (Janssen et al. 2001), and they have produced support for a causal association between traffic-related air pollution and respiratory and allergic outcomes in children (Brauer et al. 2007; Gauderman et al. 2005, 2007; Gehring et al. 2002). Also, experimental evidence suggests that diesel particles may enhance allergic sensitization to common inhalant allergens (Diaz-Sanchez 1997; Diaz-Sanchez et al. 1997; Li et al. 2009; Nel et al. 1998).

Most of the studies conducted to date were from developed countries. One exception is a study from Ethiopia (Venn et al. 2005), which also found a relationship between wheeze and distance from home to a busy street. As car and truck traffic increases in the world, study of a wider range of countries is needed. In this article, we present findings on truck traffic exposure from the third phase of the International Study of Asthma and Allergies in Childhood (ISAAC), a questionnaire-based assessment that was conducted in > 1,187,000 children from 238 centers located in 98 countries in all parts of the world. The analyses in this article are restricted to 513,087 children for whom self-reported truck traffic exposure data were collected.

## Materials and Methods

ISAAC phase 3 is a repetition and expansion of the first phase of ISAAC, which documented large differences in the prevalence of childhood allergic symptoms across the world (Ellwood et al. 2005; ISAAC 1998).

As in ISAAC phase 1, written questionnaires were self-completed at school by 13- to 14-year-olds as well as, in most centers, a video questionnaire on wheezing symptoms. Parents of the 6- to 7-year-olds completed the written questionnaire at home. Schools were randomly selected from within a defined geographic area. Centers obtained ethical approval from their local ethics committees or, for the minority of centers that did not have an ethics committee, some other approving body such as the ministry of health. The method of consent was determined by the local ethics committee, and centers obtained their own funding. Adherence to the ISAAC protocol was assessed, and centers with serious discrepancies were excluded. Minor deviations were footnoted (Asher et al. 1998, 2006; Strachan et al. 1997; Williams et al. 1999). In this article, we focus on “current wheeze” [“Have you (Has your child) had wheezing or whistling in the chest in the past 12 months?”], “asthma ever” [“Have you (Has your child) ever had asthma?”], symptoms of “rhinoconjunctivitis” [“In the past 12 months, have you (has your child) had a problem with sneezing, or a runny, or blocked nose when you (he/she) DID NOT have a cold or the flu?” and “In the past 12 months, has this nose problem been accompanied by itchy-watery eyes?”], and symptoms of “eczema” [“Have you (Has your child) had this itchy rash at any time in the past 12 months?” and “Has this itchy rash at any time affected any of the following places: the folds of the elbows, behind the knees, in front of the ankles, under the buttocks, or around the neck, ears or eyes?”]. These questions were preceded by the question “Have you (Has your child) ever had an itchy rash coming and going for at least 6 months?” We have also analyzed “symptoms of severe asthma,” defined as those with current wheeze who, according to the written questionnaire, in the past 12 months have had four or more attacks of wheeze, or one or more nights of sleep disturbance from wheeze per week, or wheeze affecting speech. This definition is based on previous ISAAC analyses that showed that a combination of these features of more severe wheezing episodes correlated significantly more closely with asthma mortality and hospital admissions than did current wheeze alone (Anderson et al. 2008). In addition, the 13- to 14-year-olds were asked to respond to a video questionnaire showing various symptoms of wheeze in children of similar age, and a positive response to the question relating to the scene of the young person wheezing at rest “Has your breathing ever been like this in the past 12 months?” was defined as “current wheeze–video” (Crane et al. 1995). In ISAAC phase 3, an optional environmental questionnaire (EQ) was administered in addition to the symptom questionnaire to test a number of specific etiologic hypotheses (Ellwood et al. 2005). The EQ included questions on diet, heating and cooking fuels, exercise, pets, family size, birth order, socioeconomic status, use of antibiotics and antipyretics, breast-feeding, birth weight, immigrant status, environmental tobacco smoke, and frequency of truck traffic on the street of residence. The complete questionnaire can be found on the ISAAC Web site (ISAAC 2009). The question that this article addresses is “How often do trucks pass through the street where you live, on weekdays?” The four answers to choose from were never, seldom, frequently through the day, and almost the whole day.

For this article, we compared children in the “almost the whole day,” “frequently through the day,” and “seldom” categories, respectively, with children in the “never” category as baseline in order to explore the existence of an exposure–response relationship. We calculated odds ratios (ORs) using generalized linear mixed models (GLMMs) for a binomial distribution and logit link and with the centers modeled as a random effect. The analyses on all study participants were adjusted for sex, region of the world, language, and gross national income (GNI). Regions of the world were Africa, Asia-Pacific, eastern Mediterranean, Latin America, North America, Northern and Eastern Europe, Oceania, Indian subcontinent, and Western Europe. The written questionnaire was translated from English, according to the ISAAC phase 3 protocol (Ellwood et al. 2005), into the local language(s): Arabic, Chinese, English, Hindi, Indonesian, Portuguese, Spanish, and “other” comprising many different languages. These were back-translated to English and assessed (Ellwood et al. 2009). Using GNI, centers were allocated to four categories of socioeconomic status based on their country’s GNI per capita: low, lower middle, upper middle, and high (World Bank 2006). All analyses were conducted separately for 13- to 14-year-olds and 6- to 7-year-olds. In addition to the combined analyses, further analyses were conducted after stratification for sex, region of the world, language, and GNI. Finally, multivariate analyses (GLMM) were conducted to check whether associations between symptoms and frequency of truck traffic were confounded by certain other variables in the EQ, maternal education, cooking fuel, maternal and paternal smoking, television watching, exercise, siblings (older and younger), and fast food consumption, and paracetamol (acetaminophen) use. Centers were treated as simple random effects, but region was included in the model as a fixed effect to account for the differences in level between regions.

We also investigated whether “eczema without wheeze” was associated with truck traffic. Furthermore, we investigated the effect of missing values on the findings by repeating some of these analyses using multiple imputation. We did not impute data for main exposure or outcome variables. The imputation method consisted of *a*) using the Markov chain Monte Carlo method to impute missing data to create a monotonic missing pattern and then *b*) using a logistic model to complete the imputations. All imputation was done within center.

For the adolescent group, data for 242 centers in 98 countries with 814,837 participants, and for the children, 165 centers in 65 countries with 421,543 participants, were submitted to the ISAAC International Data Centre (IIDC) for data analyses. Adherence to the ISAAC protocol was assessed, and centers with serious deviations from protocol (< 70% response rate for the adolescents and < 60% for the children, and centers with < 1,000 participants for both age groups) were excluded from the worldwide data analyses (9 centers in 6 countries with 16,152 adolescents; and 21 centers in 16 countries with 32,732 children). Centers with minor deviations from protocol were included in the analyses and identified by the use of a footnote in the ISAAC publications (Ait-Khaled et al. 2009; Asher et al. 2006; Lai et al. 2009). The final worldwide data set comprised 233 centers in 97 countries with 798,684 adolescents and 144 centers in 61 countries with 388,812 children. Centers that had not undertaken the EQ were then excluded from the data set (111 centers in 59 countries with 437,086 adolescents and 69 centers in 38 countries with 168,404 children), leaving a final EQ data set of 122 centers in 54 countries with 361,598 adolescents and 75 centers in 32 countries with 220,408 children. For inclusion in this analysis, centers were required to have ≥ 70% of participants with data on reported truck traffic exposure. Ten centers for the 13- to 14-year age group and five centers for the 6- to 7-year age group were accordingly excluded. A further two centers were excluded for the 13- to 14-year age group because of use of an invalid version of the truck exposure question.

We compared ORs between centers that were included in and excluded from the final analysis and found no systematic difference between the two.

Informed consent was obtained from parents of all participating children, and the study was approved by local institutional review boards in all participating centers.

## Results

**Figure 1:**
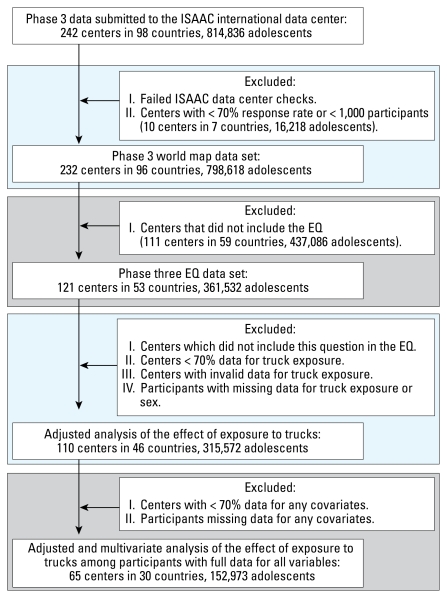
Flow chart of participation of 13- to 14-year-olds.

**Figure 2:**
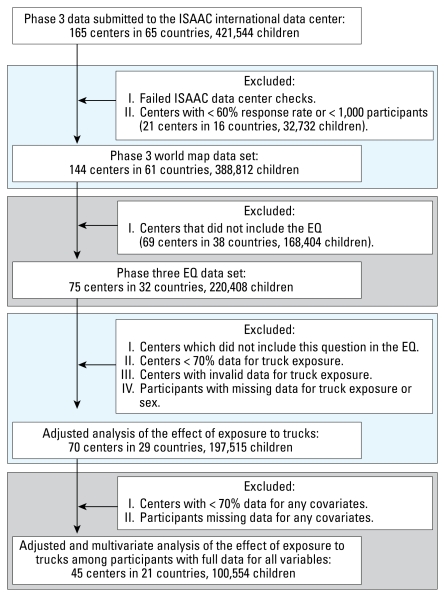
Flow chart of participation of 6- to 7-year-olds.

**Figure 3:**
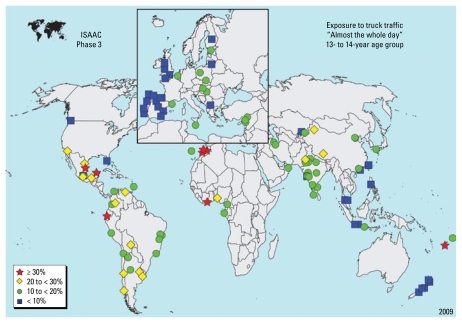
Participating centers with an indication of reported frequency of truck traffic on the street of residence: “almost the whole day” for 13- to 14-year-olds.

There were 315,572 children 13–14 years of age from 110 centers in 46 countries and 197,515 children 6–7 years of age from 70 centers in 29 countries included in the analyses. Figures 1 and 2 show a flow chart of numbers of children who were included in particular analyses. Tables 1 and 2 show the range of reported percentages of truck traffic exposure by area of the world for each age group. Somewhat surprisingly, the highest reported percentages for “high truck traffic density” were from Africa and Latin America. Reports for children 13–14 years of age by center are shown in Figure 3.

Tables 3 and 4 show the association between truck traffic on the street of residence and respiratory and allergic symptoms that were investigated in the two age groups. For all symptoms and both age categories, we found a highly significant exposure–response relationship between self-reported truck traffic frequency on the street of residence and respiratory and allergic symptoms. In both age categories, the strongest relation was for symptoms of severe asthma. Restriction of the analysis to centers with ≥ 70% availability of data on covariates, and subsequent adjustment for maternal and paternal smoking, cooking fuel, maternal education, television watching, exercise, siblings (older and younger), fast food consumption, and paracetamol use did not change these findings. Supplemental Material, Figure 1 (doi:10.1289/ehp.0800467.S1 via http://dx.doi.org) shows ORs for individual centers that were included in and excluded from the final analysis. There was no systematic difference between the two. Results for associations between environmental covariates and outcomes will be published elsewhere. In a sensitivity analysis we analyzed “eczema without wheeze,” but results were essentially similar to those for all children with eczema [Supplemental Material, Table 1 (doi:10.1289/ehp.0800467.S1)]. In another sensitivity analysis, we used an alternative specification of the cooking fuel variable contrasting “open fires” versus all others. This also did not influence the findings for self-reported truck traffic. The multiple imputation analyses that maintained the participants with missing data on the covariates also produced estimates very similar to those reported in Tables 3 and 4.

Tables 5 and 6 show the association between current wheeze and truck traffic exposures as found in different regions of the world. We found a significant exposure–response relationship in practically all regions of the world (fully adjusted results are shown only). The findings were similar for the other symptoms that were studied [Supplemental Material, Tables 2–5 (doi:10.1289/ehp.0800467.S1)]. We found no difference between boys and girls, and few differences between children from countries with different income levels (results for wheeze in 13- to 14-year-olds shown as an example in Table 7). If there was any difference, the association was weakest in the highest-income countries. Finally, we removed the three centers with the highest and the three centers with the lowest reported high truck traffic frequencies from the analyses and found no difference in results (data not shown).

**Figure 4:**
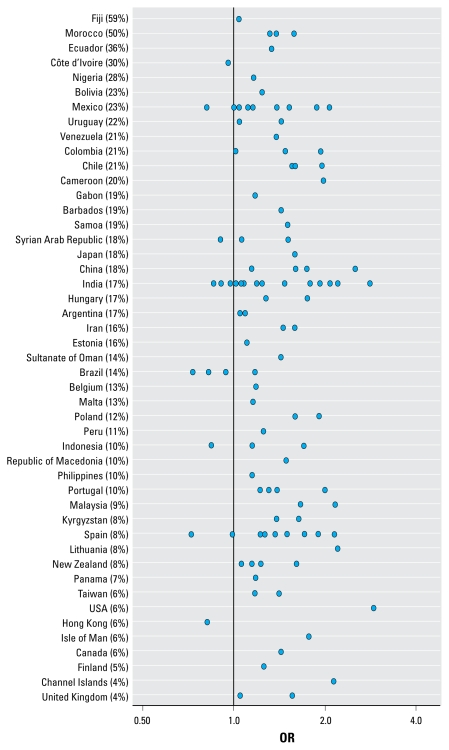
ORs of wheeze in 13- to 14-year-olds for “almost the whole day” truck traffic versus “never,” by center and country. Percentages shown are for “almost the whole day.”

**Figure 5:**
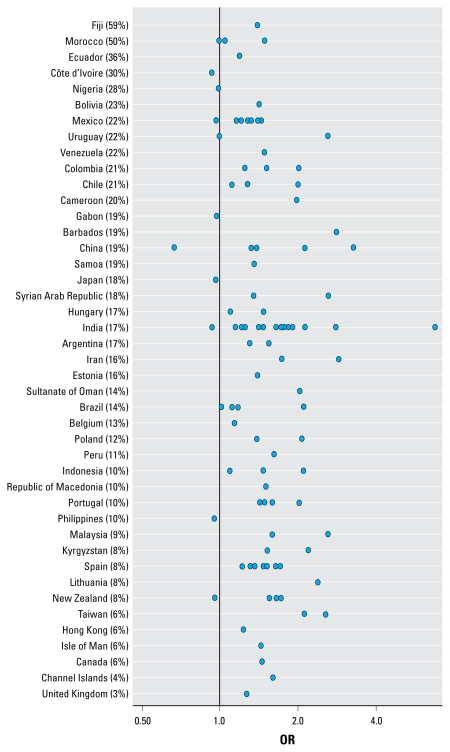
ORs of rhinoconjunctivitis in 13- to 14-year-olds for “almost the whole day” truck traffic versus “never,” by center and country. Percentages shown are for “almost the whole day.”

**Figure 6:**
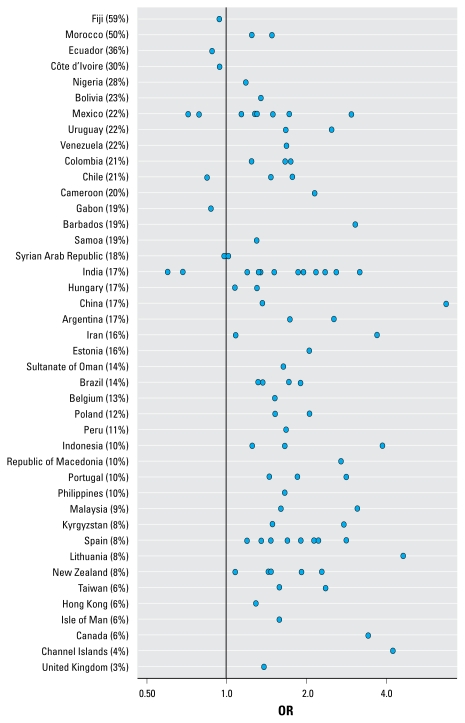
ORs of eczema in 13- to 14-year-olds for “almost the whole day” truck traffic versus “never,” by center and country. Percentages shown are for “almost the whole day.”

Figures 4, 5, and 6 show the ORs for wheeze, rhinoconjunctivitis, and eczema, respectively, in 13- to 14-year-olds for “almost the whole day” truck traffic versus “never,” by center and country. These figures show that in the large majority of centers, there was a positive association between self-reported truck traffic in street of residence and allergic outcomes.

## Discussion

In this study we found a positive global relationship between childhood symptoms of current asthma, rhinoconjunctivitis, and eczema and self-reported frequency of truck traffic on the street of residence. The associations were remarkably similar in different parts of the world, in the two age groups that were studied, and when using three different instruments (self-completed questionnaire and video questionnaire for 13- to 14-year-old children and parent-completed questionnaire for 6- to 7-year-old children) to measure “current wheeze.”

Could these associations be attributable to bias? The information obtained on symptoms and exposure was self-reported, and it is possible that the connection between reported truck traffic frequency and reported symptoms is due to bias rather than to a “true” effect. One study from the United Kingdom suggests that overreporting of traffic density by parents of symptomatic children could be responsible for some if not all of the associations seen between self-reported traffic and respiratory symptoms in children (Kuehni et al. 2006). Another study from Norway suggested the same (Piro et al. 2008), but a similar study from Italy did not (Ciccone et al. 1998). A recent study from Italy found that self-reported traffic density in area of residence was clearly associated with nitrogen dioxide, which was 39 μg/m^3^ when self-reported traffic was “absent,” 44 μg/m^3^ when “low,” 48 μg/m^3^ when “intermediate,” and 52 μg/m^3^ when “high” (Cesaroni et al. 2008). Our study found significant positive associations between rhinitis and self-reported as well as objective measures of traffic density; however, an association with asthma was found only for the self-reported measure. With the data at hand, we cannot directly refute bias in the reporting of truck traffic and/or respiratory symptoms as an alternative explanation of the findings, but there are several arguments against this. First, there are now several published studies that used objective measures of exposure and effect and found rather similar relationships between truck traffic exposure or other measures of exposure to vehicular traffic and respiratory and allergic symptoms in children (Annesi-Maesano et al. 2007; Bayer-Oglesby et al. 2006; Brauer et al. 2002, 2006, 2007; Brunekreef et al. 1997; Ciccone et al. 1998; Gauderman et al. 2005, 2007; Gehring et al. 2002; Janssen et al. 2003)

Second, these studies were conducted mostly in Western Europe and North America, and in our present global study, the associations found in these regions were not different from those found in other parts of the world. One could argue that concern about possible adverse effects on respiratory health by traffic fumes is different in different parts of the world, so one would not expect to see a universal association if responder bias played much of a role. Third, the associations were very similar for the 13- to 14-year-olds and the 6- to 7-year-olds, despite the fact that the teenagers completed the questionnaires themselves, whereas the parents completed the questionnaires for the 6- to 7-year-olds. Finally, one could argue that the use of the video questionnaire to obtain information about wheezing symptoms in the 13- to 14-year-olds has at least to some extent made the collection of the wheezing information more objective.

We could not investigate whether parents of 13- to 14-year-old and 6- to 7-year-old children from the same families gave different answers to the truck traffic questions, because data from children in these two age categories could not be linked. Also, participating centers were not classified as urban or rural, so we could not compare findings between urban and rural centers.

We can only speculate about what factors influence the remaining heterogeneity of exposure–response relationships between participating centers. Differences in rates of sensitization and bronchial hyperresponsiveness might be one explanation, because one study (Janssen et al. 2003) found these to be effect modifiers of associations between traffic exposure and symptoms. Antioxidant intake has been found to modify respiratory effects of air pollution in one study (Romieu et al. 2007). We propose that future studies carefully assess effect modification by diet. Finally, differences in genetic makeup may also play a role, as was shown in some studies (e.g., Melén et al. 2008).

The ISAAC phase 3 EQ addressed only frequency of truck traffic on the street of residence. Not much work has been conducted to investigate the validity of such questions. One study found a positive but relatively weak association between answers to a similar question and objectively modeled concentrations of traffic-related air pollutants (Heinrich et al. 2005). How misclassification associated with asking about self-reported frequency of truck traffic would affect exposure–response relationships with symptoms remains difficult to predict based on the data reported in the literature, and readers should not be encouraged by the findings of the present study to conclude that more refined exposure variables are of no more value than self-reports.

It is impossible to separate emissions from truck traffic from other forms of traffic-related air pollution on the basis of our data. Generally, roads with high truck traffic counts will be roads that carry high loads of other vehicles, as well. Thus, most likely, although the question was about truck traffic frequency, this could be a surrogate for more general traffic density. Very few studies have been able to successfully address truck traffic and car traffic separately, and those that did have suggested that truck traffic (which is almost exclusively diesel powered in the studied environments) was more important than car traffic (Brunekreef et al. 1997; Janssen et al. 2003; van Vliet et al. 1997).

High frequencies of truck traffic on the street of residence were reported in some developing countries. We are unable to validate such reports against traffic counts, but because GNI is closely linked to car ownership and intensity of road transport, we assume that reported frequencies do not reflect the same numbers of trucks passing through the street of residence in all parts of the world. The notion of “trucks” may include pickup trucks, which are in wide use especially in developing countries. We also note that trucks in developing countries are likely to be older and less well maintained than trucks in developed nations, so emissions per truck could easily be much higher in developing country settings. There is some anecdotal evidence for this effect from a study conducted in Ethiopia (Brunekreef 2005; Venn et al. 2005). Also, one would expect more dirt roads and/or more dusty roads in developing than in developed countries. Coarse particles resuspended from dirt(y) roads may contribute to high particulate matter exposures in such circumstances.

Truck traffic on the street of residence may affect allergic responses not only through air pollutant exposure but also through indirect factors such as social deprivation, stress related to noise, or accident risk associated with busy roads (Briggs et al. 2008; Chen et al. 2008). We have no means of addressing these possibilities with the data at hand. Traffic exhaust is thought to affect allergy and asthma through the inflammatory and adjuvant potential of several of its components (Brunekreef and Holgate 2002; Diaz-Sanchez 1997; Diaz-Sanchez et al. 1997; Nel et al. 1998).

A surprising finding is that associations were found between truck traffic frequency and all three allergic symptoms: wheeze, rhinoconjunctivitis, and eczema. In most studies conducted to date, eczema had not been looked at or was used only as an adjustment variable (Nicolai et al. 2003). However, some earlier studies have reported positive associations between truck traffic density or other measures of traffic-related air pollution and childhood eczema (Janssen et al. 2003; Ring et al. 1999); one study found a positive association between objectively measured air pollution from traffic and sensitization to milk and egg in a birth cohort when the children were 4 years of age (Brauer et al. 2007). Milk and egg sensitization in early childhood are typically associated with eczema. In a sensitivity analysis, we analyzed “eczema without wheeze,” but results were essentially similar to those for all children with eczema.

There is experimental evidence to support that diesel particles may enhance allergic sensitization to common inhalant allergens (Diaz-Sanchez 1997; Diaz-Sanchez et al. 1997; Nel et al. 1998). Perhaps air pollution exposure is capable of systemic immune modulation, which may then manifest as increased sensitization to food allergens commonly associated with atopic eczema. This argues for a real effect rather than one caused by reporting bias because there is no popular belief linking eczema to air pollution. Finally, a recent cohort study did find an association between traffic-related pollution and eczema at 4 years of age in children followed from birth (Morgenstern et al. 2008), and a study from Taiwan also reported a positive association between air pollution exposure and eczema in children (Lee et al. 2008).

## Conclusion

This study documents a global association between reported truck traffic frequency on the street of residence and symptoms of asthma, rhinoconjunctivitis, and eczema in children. Vehicular traffic is on the increase worldwide, with especially strong increases in such rapidly developing countries as China and India (Walsh 2007). As a result, allergic symptoms and asthma may become more prevalent in exposed populations.

## Figures and Tables

**Table 1:** Frequency of reported truck traffic density percentages in the various regions of the world where 13- to 14-year-old children were studied as part of the ISAAC phase 3 study.

	No.			Percent^a^			
	Country	Center	Total	High	Medium	Low	Never
All centers	46	110	315,572	15.9	23.7	42.8	17.6
Africa	5	7	16,914	31.6	20.5	28.7	19.2
Asia-Pacific	7	15	49,707	12.5	20.6	45.2	21.7
Eastern Mediterranean	4	7	22,745	16.2	24.9	49.4	9.5
Indian subcontinent	1	16	43,298	17.2	22.0	35.4	25.4
Latin America	10	27	76,375	20.7	23.5	38.5	17.3
North America	3	3	6,452	10.9	24.1	49.9	15.1
Northern and Eastern Europe	7	10	30,026	11.6	23.7	55.0	9.7
Oceania	3	7	19,002	17.8	27.1	39.5	15.6
Western Europe	6	18	51,053	7.9	27.7	47.9	16.5

a High, almost the whole day; medium, frequently during the day; low, seldom.

**Table 2:** Frequency of reported truck traffic density percentages in the various regions of the world where 6- to 7-year-old children were studied as part of the ISAAC phase 3 study.

	No.			Percent^a^			
	Country	Center	Total	High	Medium	Low	Never
All centers	29	70	197,515	14.6	24.4	41.0	20.0
Africa	1	1	2,211	20.4	23.0	32.6	24.0
Asia-Pacific	5	8	26,860	12.8	19.6	40.5	27.1
Eastern Mediterranean	4	6	18,741	15.3	25.6	47.5	11.6
Indian subcontinent	1	14	41,872	15.7	22.2	35.5	26.6
Latin America	6	16	43,314	22.2	23.8	35.4	18.6
North America	2	2	3,881	12.0	21.9	41.0	25.1
Northern and Eastern Europe	5	6	15,103	14.3	26.0	48.0	11.7
Oceania	1	4	10,618	6.7	26.4	51.1	15.8
Western Europe	4	13	34,915	7.3	29.9	45.8	17.0

a High, almost the whole day; medium, frequently during the day; low, seldom.

**Table 3:** Association between self-reported truck traffic on the street of residence and symptoms in 13- to 14-year-old children.

	No.			OR (95% confidence interval)		
Group/symptom	Country	Center	Total	High vs. never	Medium vs. never	Low vs. never
All study participants^a^						
Current wheeze	46	110	315,572	1.46 (1.36–1.56)	1.33 (1.25–1.41)	1.13 (1.07–1.19)
Asthma ever	46	110	315,572	1.23 (1.16–1.30)	1.11 (1.05–1.17)	1.04 (0.99–1.09)
Current wheeze–video	35	86	246,658	1.53 (1.40–1.67)	1.35 (1.25–1.47)	1.13 (1.04–1.22)
Severe asthma symptoms	46	108	310,808	1.65 (1.53–1.79)	1.35 (1.26–1.46)	1.10 (1.03–1.18)
Rhinoconjunctivitis	46	110	315,572	1.49 (1.41–1.59)	1.28 (1.21–1.35)	1.09 (1.03–1.14)
Eczema	45	109	313,085	1.59 (1.47–1.72)	1.35 (1.25–1.44)	1.09 (1.03–1.17)

Study participants with complete confounder data^a^,^b^						
Current wheeze	30	65	152,973	1.47 (1.33–1.62)	1.31 (1.20–1.43)	1.09 (1.00–1.18)
Asthma ever	30	65	152,973	1.24 (1.14–1.35)	1.12 (1.04–1.21)	1.02 (0.95–1.10)
Current wheeze–video	26	52	121,930	1.58 (1.39–1.79)	1.35 (1.20–1.52)	1.13 (1.01–1.26)
Severe asthma symptoms	30	63	149,488	1.72 (1.53–1.93)	1.37 (1.22–1.52)	1.09 (0.99–1.21)
Rhinoconjunctivitis	30	65	152,973	1.51 (1.38–1.65)	1.29 (1.19–1.40)	1.08 (1.00–1.16)
Eczema	30	65	152,973	1.67 (1.49–1.87)	1.37 (1.23–1.52)	1.10 (0.99–1.21)

Study participants with complete confounder data, adjusted for potential confounders^a^,^b^,^c^						
Current wheeze	30	65	152,973	1.35 (1.23–1.49)	1.24 (1.13–1.35)	1.07 (0.98–1.16)
Asthma ever	30	65	152,973	1.18 (1.08–1.28)	1.08 (1.00–1.17)	1.01 (0.94–1.09)
Current wheeze–video	26	52	121,930	1.44 (1.26–1.64)	1.28 (1.14–1.44)	1.11 (0.99–1.23)
Severe asthma symptoms	30	63	149,488	1.53 (1.36–1.72)	1.26 (1.13–1.41)	1.07 (0.97–1.18)
Rhinoconjunctivitis	30	65	152,973	1.39 (1.27–1.52)	1.21 (1.12–1.32)	1.06 (0.98–1.14)
Eczema	30	65	152,973	1.54 (1.37–1.73)	1.30 (1.17–1.45)	1.08 (0.97–1.19)

a Adjusted for sex, region of the world, language, and GNI.

b Centers included if ≥ 70% data were available for all covariates; analysis was restricted to those with complete data for all covariates.

c Also adjusted for maternal education, cooking fuel, maternal and paternal smoking, television watching, exercise, siblings (older and younger), fast-food consumption, and paracetamol use.

**Table 4:** Association between self-reported truck traffic on the street of residence and symptoms in 6- to 7-year-old children.

	No.			OR (95% confidence interval)		
Group/symptom	Country	Center	Total	High vs. never	Medium vs. never	Low vs. never
All study participants^a^						
Current wheeze	29	70	197,515	1.46 (1.36–1.56)	1.31 (1.24–1.39)	1.09 (1.03–1.15)
Asthma ever	29	70	197,515	1.32 (1.23–1.42)	1.21 (1.14–1.28)	1.04 (0.98–1.10)
Severe asthma symptoms	29	69	194,932	1.64 (1.49–1.80)	1.33 (1.22–1.45)	1.08 (1.00–1.17)
Rhinoconjunctivitis	29	70	197,515	1.44 (1.34–1.54)	1.24 (1.17–1.32)	1.07 (1.01–1.13)
Eczema	28	69	194,622	1.37 (1.28–1.48)	1.20 (1.13–1.28)	1.08 (1.02–1.14)

Study participants with complete confounder data^a^,^b^						
Current wheeze	21	45	100,554	1.48 (1.34–1.63)	1.35 (1.25–1.46)	1.08 (1.01–1.16)
Asthma ever	21	45	100,554	1.28 (1.16–1.42)	1.19 (1.10–1.28)	1.02 (0.95–1.10)
Severe asthma symptoms	21	45	100,554	1.65 (1.45–1.89)	1.35 (1.21–1.51)	1.07 (0.97–1.19)
Rhinoconjunctivitis	21	45	100,554	1.43 (1.29–1.59)	1.20 (1.10–1.30)	1.01 (0.93–1.09)
Eczema	21	45	100,554	1.42 (1.29–1.56)	1.21 (1.11–1.31)	1.08 (1.00–1.16)

Study participants with complete confounder data, adjusted for potential confounders^a^,^b^,^c^						
Current wheeze	21	45	100,554	1.35 (1.22–1.48)	1.27 (1.17–1.38)	1.05 (0.98–1.13)
Asthma ever	21	45	100,554	1.20 (1.09–1.33)	1.13 (1.05–1.23)	1.00 (0.93–1.08)
Severe asthma symptoms	21	45	100,554	1.41 (1.23–1.62)	1.23 (1.10–1.38)	1.03 (0.93–1.14)
Rhinoconjunctivitis	21	45	100,554	1.33 (1.20–1.48)	1.14 (1.05–1.24)	0.99 (0.91–1.07)
Eczema	21	45	100,554	1.36 (1.23–1.50)	1.18 (1.09–1.28)	1.07 (0.99–1.15)

a Adjusted for sex, region of the world, language, and GNI.

b Centers were included if ≥ 70% data available for all covariates; analysis was restricted to those with complete data for all covariates.

c Also adjusted for maternal education, cooking fuel, maternal and paternal smoking, television watching, exercise, siblings (older and younger), fast food consumption, and paracetamol use.

**Table 5:** Adjusted^a^ association between self-reported truck traffic on the street of residence and current wheeze in 13- to 14-year-old children participating in the ISAAC phase 3 study in different parts of the world.

	No.			OR (95% confidence interval)^b^		
Region	Country	Center	Total	High vs. never	Medium vs. never	Low vs. never
Africa	2	2	2,656	1.30 (0.57–2.95)	1.13 (0.43–2.92)	0.91 (0.34–2.40)
Asia-Pacific	6	10	29,271	1.23 (0.94–1.61)	1.28 (1.03–1.59)	1.09 (0.91–1.31)
Eastern Mediterranean	2	4	8,700	1.43 (0.76–2.68)	1.26 (0.69–2.32)	1.11 (0.62–2.00)
Indian subcontinent	1	9	19,029	1.49 (1.01–2.20)	1.49 (1.03–2.15)	1.23 (0.87–1.74)
Latin America	10	18	40,300	1.38 (1.17–1.64)	1.27 (1.08–1.50)	1.08 (0.93–1.27)
North America	2	2	4,525	1.71 (0.91–3.20)	1.58 (0.93–2.68)	1.52 (0.94–2.45)
Northern and Eastern Europe	5	7	18,659	1.44 (1.08–1.90)	1.30 (1.01–1.69)	1.06 (0.83–1.35)
Western Europe	2	13	29,833	1.22 (0.98–1.53)	1.06 (0.89–1.26)	0.94 (0.80–1.11)

a Adjusted for sex, language, GNI, maternal education, maternal and paternal smoking, exercise, television watching, fast food consumption, current paracetamol use, siblings, and cooking fuel.

b High, almost the whole day; medium, frequently during the day; low, seldom.

**Table 6:** Adjusted^a^ association between self-reported truck traffic on the street of residence and current wheeze in 6- to 7-year-old children participating in the ISAAC phase 3 study in different parts of the world.

	No.			OR (95% confidence interval)^b^		
Region	Country	Center	Total	High vs. never	Medium vs. never	Low vs. never
Africa	1	1	833	1.41 (0.24–8.43)	0.32 (0.03–3.32)	0.32 (0.04–2.71)
Asia-Pacific	3	5	13,064	1.27 (0.95–1.69)	1.25 (0.98–1.61)	0.93 (0.75–1.15)
Eastern Mediterranean	2	4	7,882	1.20 (0.83–1.73)	1.12 (0.79–1.59)	0.93 (0.67–1.28)
Indian subcontinent	1	7	16,972	2.43 (1.66–3.56)	1.41 (0.97–2.04)	1.48 (1.06–2.07)
Latin America	5	7	15,641	1.51 (1.22–1.88)	1.33 (1.09–1.61)	1.08 (0.91–1.30)
North America	2	2	3,076	1.60 (1.12–2.29)	1.88 (1.37–2.59)	1.13 (0.87–1.48)
Northern and Eastern Europe	3	3	6,592	1.12 (0.76–1.66)	1.23 (0.87–1.74)	0.98 (0.71–1.36)
Oceania	1	4	9,541	1.08 (0.83–1.41)	1.16 (0.96–1.39)	1.02 (0.87–1.21)
Western Europe	3	12	26,953	1.23 (1.00–1.52)	1.22 (1.06–1.41)	1.07 (0.93–1.22)

a Adjusted for sex, language, GNI, maternal education, maternal and paternal smoking, exercise, television watching, fast food consumption, current paracetamol use, siblings, and cooking fuel.

b High, almost the whole day; medium, frequently during the day; low, seldom.

**Table 7:** Adjusted^a^ association between self-reported truck traffic on the street of residence and current wheeze in 13- to 14-year-old children participating in the ISAAC phase 3 study in countries in different income categories.

	No.		OR (95% confidence interval)^b^		
Income level	Country	Center	High vs. never	Medium vs. never	Low vs. never
High	5	17	1.26 (1.04–1.52)	1.13 (0.98–1.31)	1.01 (0.89–1.16)
Upper middle	11	18	1.47 (1.23–1.74)	1.31 (1.11–1.54)	1.12 (0.95–1.31)
Lower middle	12	20	1.33 (1.10–1.60)	1.29 (1.08–1.54)	1.06 (0.89–1.25)
Low	2	10	1.45 (1.00–2.11)	1.45 (1.02–2.07)	1.21 (0.87–1.69)

a Adjusted for sex, language, GNI, maternal education, maternal and paternal smoking, exercise, television watching, fast food consumption, current paracetamol use, siblings, and cooking fuel.

b High, almost the whole day; medium, frequently during the day; low, seldom.
